# Repeated Sprint Ability Demands in U16 to U19 Highly Trained Handball Players Concerning Playing Position

**DOI:** 10.3390/ijerph17165959

**Published:** 2020-08-17

**Authors:** Michal Starczewski, Lech Borkowski, Piotr Zmijewski

**Affiliations:** Department of Physiology, Institute of Sport—National Research Institute, Trylogii 2/16, 01-982 Warsaw, Poland; lech.borkowski@insp.waw.pl (L.B.); piotr.zmijewski@insp.waw.pl (P.Z.)

**Keywords:** youth handball players′ characteristics, RSA test, anaerobic performance, playing position characteristics

## Abstract

The aim of the study was to determine anaerobic capacity and characterize changes in repeated sprint ability (RSA) within youth elite handball players. For this study, 142 male athletes (17.1 ± 0.9 years) were recruited from a handball sports high school and performed the RSA test on a cycle ergometer, including five 6 s all-out efforts separated by 24 s passive breaks. Maximal (P_max_) and mean (P_mean_) power, highest (W_max_), and total work (W_tot_) as well as power (P_dec_) and work (W_dec_) decrement were measured. Significant differences in RSA were noted in relation to age (greater values of P_max_, P_mean_, W_tot_, W_dec_, and P_dec_ in U19 than U17 as well as greater values of P_max_, W_tot_, W_max_, W_dec_, and P_dec_ in U19 than U16 (*p* < 0.05)) and playing position (wing players had greater W_tot_ than pivot, 269 vs. 243 (J/kg) (*p* < 0.05), and wing players differed significantly in absolute and relative power from athletes of other positions). RSA depends on playing position and age in groups of youth handball players and the RSA test can be helpful in the selection of athletes for a playing position. The article introduces normative values for elite youth handball players, empowering coaches in the evaluation of anaerobic abilities and selection.

## 1. Introduction

The repeated sprint ability (RSA) test is widely applied in team and racket sports. Handball, soccer, basketball, and tennis players are expected to generate in intervals high values of muscle power (sprints) in order to respond to specific playing situations, alternated by periods of low physical activity [1,2]. Girard et al. (2011) noted that the time dedicated to sprint in team sports accounts for 1 to 4% of the effective playtime. However, the ability to develop high values of power and to maintain it during several subsequent actions in a game can be crucial for success [3].

Within the RSA test maximum values of variables defining the ability to repeat sprints are measured. With regard to running tests this accounts for the time needed to cover the distance and, in the case of tests performed on a cycle ergometer, the maximum power. The fatigue index is measured as the percentage speed decrease in the subsequent part of the running distance and the percentage decrease in power or work in repeated trials performed on a cycle ergometer [3].

In studies carried out on elite youth handball players by Ingebrigtsen et al. (2013) [4], a difference related to the age and experience of subjects was found in results achieved in running RSA tests conducted on U18 and U16 players. On the other hand, Buchheit et al. (2010) [5] evaluated the impact of training on sprint abilities measured in RSA tests in 14 young handball players. That observation confirmed that this ability is trainable in youth, and specifically for handball. The dependence between the physiological profile of handball players and their playing position as well as their sport level has been examined [6]. Authors reached the conclusion that a dependence between body composition, physical performance abilities, and playing position of handball players from the 1st and 2nd German League can be found [6]. Another interesting issue related to repeated sprint ability is the differences in results between wings (W), pivot (P), goalkeepers (G), and back (B) players. Previous research conducted on handball players investigated the anthropometric profile and physiological tests of 181 female youth players. The results showed that wings had better performance than all other players in the broad jump, 30 m sprint, and VO_2_max [7]. In the other study, conducted on 53 elite female handball players, only the 30 m sprint test was a factor that determined differences between playing positions [8]. The playing position is also related to shot-specific muscle strength and power as well as injury incidents in male players [9,10]. Moreover, papers describing the impact of cortisol and testosterone on performance in team sports [11] and theirs responses to training regardless of playing position in handball suggest that G have a greater hormonal reactivity than other players [12]. Differences in RSA resulting from playing positions have been observed in research conducted in other team sports. The research conducted on 120 soccer players showed differences in the sprint time between defenders, midfielders, and forwards [13]. Moreover, research performed on 110 professional basketball players led to the conclusion that the running jumps have discriminative validity in differentiating playing positions in basketball [14]. From this brief literature overview, it is clear that the playing position has an impact on performance in handball and other team sports. Evidently, little is known about the validity of RSA test results and their applicability in differentiating between playing positions in youth male handball players.

The complexity of handball preparation, as well as the importance of RSA test results for practice, was presented in the previous mentioned publications. Those findings led us to investigate the dynamics of changes in the measured RSA variables in U16 to U19 groups. To date, the dynamics of changes in RSA have not been investigated in such broad age groups of youth handball players. The monitoring of athletes from different youth age groups seems to be crucial to determine the predictive utility of the applied test, and the understanding of the factors that contribute to handball performance. One of the aims of preseason training is to improve RSA and for this reason the normative values could help to optimize training loads. Therefore, the aim of the study was to determine changes in repeated sprint ability between players from identified age groups (U16, U17, U18, and U19) and various playing positions.

## 2. Materials and Methods

### 2.1. Participants

For this study, 142 male handball players with training experience of more than 3 years at the age of 17.1 ± 0.9 years (9 in the age of 15–15.9 years (U16), 60 in the age of 16–16.9 years (U17), 39 in the age of 17–17.9 years (U18), and 34 in the age of 18–18.9 years (U19)) were recruited from handball sport high schools. Players were selected to the study by coaches, which affected the numbers in the chosen age groups. The athletes participated voluntarily and were informed about the right to withdraw at every stage of the research. All participants provided written consent for participation, and in the case of underage players, such consent was received from their guardians. The research protocol was approved by the ethics commission at the Institute of Sport–National Research Institute in Warsaw, Poland (KEBN-17-32-KB), in compliance with the Declaration of Helsinki.

### 2.2. Procedures

Although the RSA tests in handball players have so far been conducted within running trials [15,16,17], in the presented study the cycle ergometry test was used. The cycle ergometry RSA test is more reliable than the running test and is focused on the physiological aspects of lower limb power and speed/force features. It also allowed us to evaluate other aspects of the RSA than running tests, which was described in publications of the other authors [2,18,19]. The method used to determine total work and the decrement (%) in power and work have been described previously [20]. The cycling protocol provides both a valid and reliable test of RSA [21] with coefficient of variation 3.7% in recreationally active female students [20]. Moreover, cycle ergometry tests have often been used before in order to determine anaerobic capacity in handball players [17,22,23,24] as well in the RSA test [1,25,26].

In order to determine the repeated sprint ability, the RSA test was conducted on the cycle ergometer Monark 874E. The ambient temperature for testing sessions ranged from 18 to 22 °C and tests were performed at the same time of day, between 10.00 AM and 1.00 PM for all subjects. Players were instructed not to perform intense exercise on the day before a test and to consume their last meal at least 2 h before the scheduled test time. The RSA test was composed of five 6 s intervals of maximal effort alternated with 24 s of passive rest. During the test, the athletes were instructed to reach the greatest possible cadence of weight-bearing pedaling (friction weight accounted for 7.5% of the body mass) in each interval. Following a 5 min warm up (about 70 W) with two 3 s sprints between, each athlete was allowed a 5 min recovery before performing the test. All sprints were performed from a stopped position and passive recovery was utilized between sprints. Five seconds before starting the next sprint, the athletes were asked to keep a ready position and wait for the start.

The maximal power and work values were calculated from the best 6 s interval. An absolute (total kJ and W) and relative (J/kg and W/kg) work and power score was calculated along with their respective fatigue index (% of decrement over repeated efforts). The equations for the fatigue index calculation were as previously described by Orysiak et al. (2018) [27].

### 2.3. Decrement of Work and Power

Decrement of work

W_dec_ = 100 − (W_tot_·(W_max_∙5)^−1^·100)

W_dec_—decrement of work, W_tot_—total work, W_max_—highest 6 s work.

Decrement of power

P_dec_ = 100 − (P_min_·P_max_^−1^·100)

P_dec_—decrement of power, P_min_—lowest 6 s power, P_max_—highest 6 s power.

### 2.4. Statistical Analysis

All results were presented as mean and standard deviation. Prior to the statistical analysis, tests for normality (Kolmogorov–Smirnov and Shapiro–Wilk) and a test for the homogeneity of variance (Levene’s) were carried out on all variables. In order to determine the differences in RSA variables between age groups and playing position, the Kruskal–Wallis test and Bonferroni post hoc test for multiple comparisons were applied. The level of significance was set at *p* < 0.05. All calculations were performed with STATISTICA software (v. 12.0, StatSoft, TIBCO Software Inc., Palo Alto, CA, USA).

## 3. Results

In Table 1, changes in RSA test variables in U16, U17, U18, and U19 age categories are presented. The Kruskal–Wallis test did not show any significant differences in height, P_max_, W_tot_, and W_max_ relative values between the examined groups. Significant differences in the remaining variables (P_max_, P_mean_, P_mean/kg_, W_tot_, W_abs_, P_dec_, and W_dec_) were found. As a result of multiple comparison tests, significant differences of the abovementioned variables were determined with regard to the respective age groups (Table 1).

When comparing players of different positions, regardless of U16 to U19 categories, it was observed that only age and W_dec_ were not significant indicators differentiating players from the different playing position (Table 2).

**Figure 1:**
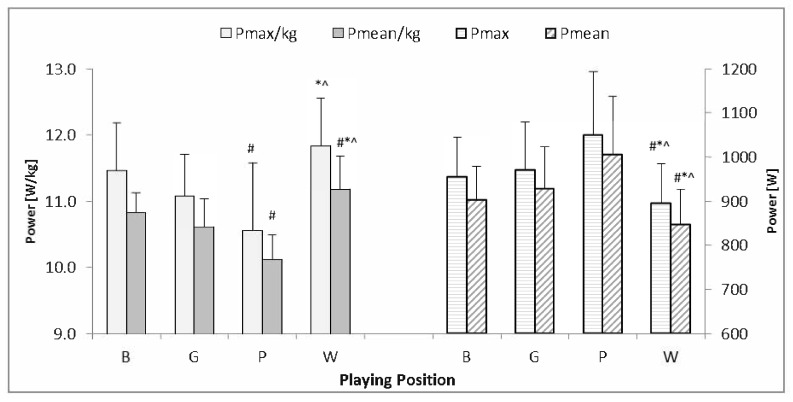
Differences in absolute maximal power (P_max_) and mean power (P_mean_) as well as relative maximal power (P_max/kg_) and mean power (P_mean/kg_) in the RSA test for athletes of various playing positions. B—backs, G—goalkeepers, P—pivots, W—wings, #—significantly different from B, *—significantly different from G, ^—significantly different from P.

The absolute power values P_max_, P_mean_ were significantly lower (*p* < 0.05) in W players than in the other groups. On the other hand, relative values of P_max_, P_mean_ were the highest in W in comparison to B, G, and P athletes (*p* < 0.05). Relative P_max_ and P_mean_ were also significantly lower in the P compared to B group (*p* < 0.05) (Figure 1).

**Figure 2:**
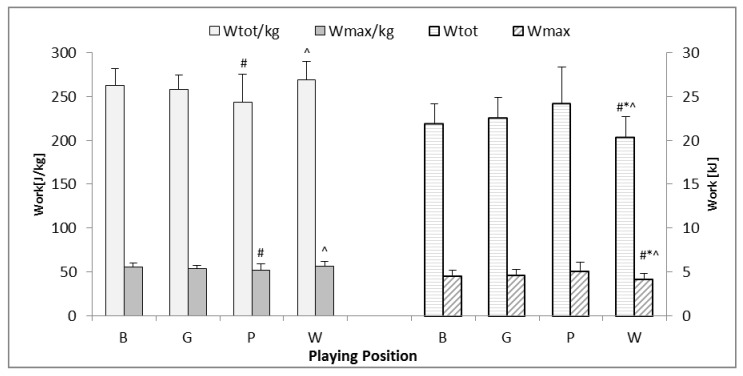
Differences in absolute maximal work (W_max_) and total work (W_tot_) as well as relative maximal work (W_max/kg_) and total work (W_tot/kg_) in the RSA test for athletes of various playing positions. B—backs, G—goalkeepers, P—pivots, W—wings, #—significantly different from B, *—significantly different from G, ^—significantly different from P.

A similar relationship to that presented in Figure 1 can be found in W_max_ and W_tot_ values. The W players had significantly lower absolute values of those variables than B, G, and P players (*p* < 0.05). However, the relative values of W_max_ and W_tot_ were higher in the W and B groups than in P (*p* < 0.05) (Figure 2).

**Figure 3:**
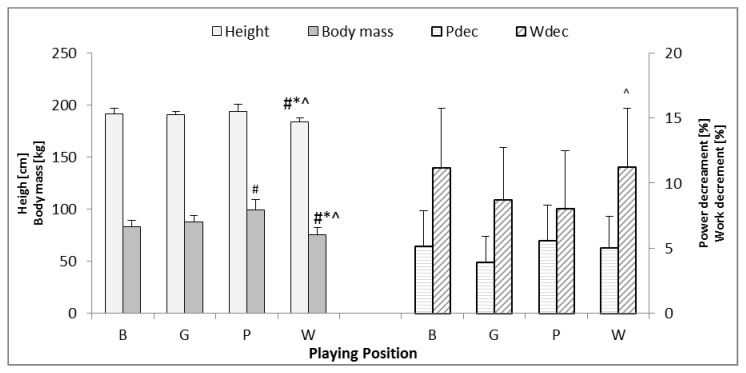
Differences in height, body mass, work decrement (W_dec_), and power decrement (P_dec_) in the RSA test for athletes of various playing positions. B—backs, G—goalkeepers, P—pivots, W—wings, #—significantly different from B, *—significantly different from G, ^—significantly different from P.

Significantly lower body mass and height were observed in wings in comparison to other players (*p* < 0.001 for all positions). On the other hand, pivots were characterized by significantly higher body mass in comparison to backs (*p* < 0.001) (Figure 3).

Only wings achieved significantly higher values of the W_dec_ indicator in respective test trials in comparison to pivot players (*p* < 0.05) (Figure 3). In other cases, no significant differences were noted with regard to fatigue variables in the RSA test (P_dec_ and W_dec_) (Figure 3).

## 4. Discussion

The main observation resulting from this research is that the values of the RSA test variables are determined by the age and playing position. The dynamics of changes in RSA variables in age categories U16 to U19 are presented. The study introduces normative values for elite youth handball players, empowering coaches in the evaluation of repeated sprint abilities and the process of selection. Significant differences between the U19 group and the younger categories in absolute values of P_max_ (*p* < 0.001), P_mean_ (*p* = 0.002), W_tot_ (*p* < 0.001), W_max_ (*p* < 0.001), P_dec_ (*p* = 0.002), and W_dec_ (*p* = 0.002) were noted (Table 1). In this study, significant differences in power and work for the majority of the measured variables were observed in players of the U19 category when compared to the U16 category (Table 1). It is highly probable that the observed changes may be related to maturity status and the physical development of the players at age 15 to 19 years. Similar observations of significant changes in maximal power with the increasing sport experience were found in a work conducted on 197 handball players [24]. The publication showed that the cadet elite players (17.3 ± 0.6 years of age) obtained significantly lower maximal power in comparison to junior elite players’ (18.6 ± 0.9 years of age) maximal power [24]. As opposed to the studies of the abovementioned author, no significant differences between groups of similar age were noted in the showed data (Table 1). The authors of this publication assume that the differences in results may have been affected by the training experience and the maturity of athletes. Regarding the sports level, the investigated players were previously selected for a handball sports school.

On the other hand, conclusions similar to the ones presented in the study were reached in the research regarding handball players in groups U14 and U16 whose age did not have any significant impact on physical performance level [28]. However, despite the fact that no significant differences in changes of P_max/kg_ (*p* = 0.421) due to age were demonstrated, a tendency to increase with age can be noted in the case of this variable (Table 1). We should also consider training impact on power. In a publication by Maroto-Izquierdo et al. (2020), the authors concluded that six weeks of pneumatic or flywheel training resulted in increases in shoulder strength and power and throwing speed [29]. Presented results also showed significant differences in the absolute values of the W_max_ variable in U19 players in comparison to other groups. That may suggest that W_max_ can have a diagnostic value in the development of repeated sprint ability in youth handball players (Table 1). The use of the W_max_ variable measured in the RSA test was not previously described in the literature, but can be an area for a further investigation.

In the described study, the anthropometric characteristics (height and body mass) differentiated W players from B, G, and P (Figure 3). However, P players had significantly greater body mass than B. It may suggest that particular playing positions require appropriate motor abilities and are determined by the dimensions of the body. A previous publication showed significantly higher values of height and body mass in backs and pivots among teams taking part in the World Seniors Championships [30]. In another work, conducted on Italian handball players, significant differences in body composition of wings and goalkeepers in comparison to athletes playing in other playing positions were observed [31]. The results achieved in this study, and by mentioned authors, may imply that the assignment of playing position should be determined by the player’s anthropometric characteristics.

The players anthropometric characteristics also had an impact on the physiological characteristics shown in Table 2. Wing players achieved the highest scores of relative values of the work and power test, but the absolute values were the lowest (Figure 1 and Figure 2). However, no significant differences of the P_dec_ and W_dec_ values were found between groups. In addition, results showed the highest absolute values of P_max_, P_mean_, W_tot_, and W_max_ in P players. Differences between wings and other players were also observed in Greek adult handball players [23]. The study of Nikolaidis et al., similarly to the results achieved in this research, showed significant differences of absolute P_max_ and P_mean_ in adult players, where the pivot players had the highest values, and wings the lowest. On the other hand, no significant differences were found in the adolescents [23]. With regard to relative values, similarly to the presented findings, adult wing players achieved the highest values of the examined anaerobic performance variables in comparison to other players. Similar dependencies of playing position related to the predictors of repeated sprint ability were found in elite female handball players [7,8]. Those findings may suggest that to monitor development of W players, focus should be on relative, and in P players on absolute, RSA values.

This study is not exempt of limitations. Firstly, the division into the age and playing position groups caused an unequal number of athletes. It was related to the Polish school system and the number of athletes on the chosen position in the team, and we had no impact on that. Secondly, we used the cycle ergometer rather than running RSA tests, which is most common in team sports testing. On the other hand, the main value of this study is that the chosen test allowed us to measure mechanical variables as power and work in large groups of athletes with high reliability.

The strength of this work is that the results indicate the relationship between age, handball playing position, and specific characteristics of anaerobic performance and anthropometric features. These differences should therefore be considered in the process of team selection and youth development. The wings are characterized by low height and body mass in comparison to other players, which result in the highest values of relative power and work.

## 5. Conclusions

The conclusion drawn from the performed analysis is that age and playing position have a significant impact on physical fitness in handball. This is evidenced by significantly higher RSA test results in players from the U19 group in comparison to other groups. The study confirms the impact of the playing position on the results achieved in the RSA test. Wing players achieved the best relative scores, whereas pivots achieved the best absolute results predicting repeated sprint ability.

Based on the test results, mean values of physiological and anthropometrical variables were established in relation to age and playing position groups. These values may become of practical use for handball coaches in the ongoing control of the athlete’s power–speed preparation in U16, U17, U18, U19 groups as well as assignment to the playing positions.

## Figures and Tables

**Table 1:** Repeated sprint ability (RSA) test results with regard to age groups.

	Unit	Age Groups				
		U16 (n = 9)	U17 (n = 60)	U18 (n = 39)	U19 (n = 34)	*p*
Height	cm	191 ± 3.48	188 ± 5.97	189 ± 6.37	190 ± 6.31	0.397
Body mass	kg	82.0 ± 8.55	81.6 ± 9.36	84.0 ± 8.90	86.6 ± 9.70 ^†^	0.041
P_max_	W/kg	11.0 ± 0.47	11.2 ± 0.81	11.5 ± 0.81	11.9 ± 0.78	0.421
	W	899 ± 77.7	906 ± 91.6	966 ± 91.0†	1024 ± 114 *^,†^	<0.001
P_mean_	W/kg	10.6 ± 0.48	10.7 ± 0.69	10.9 ± 0.65	11.1 ± 0.59	0.017
	W	864 ± 69.5	867 ± 85.4	914 ± 88.8	959 ± 107 ^†^	0.002
W_tot_	J/kg	256 ± 16.7	256 ± 22.7	262 ± 18.5	275 ± 21.0	0.394
	kJ	21.0 ± 2.81	20.8 ± 2.44	21.9 ± 2.04	23,8 ± 3.00 *^,†^	<0.001
W_max_	J/kg	52.9 ± 3.79	53.4 ± 4.92	55.3 ± 4.60	58.8 ± 5.08	0.594
	kJ	4.34 ± 0.61	4.34 ± 0.52	4.63 ± 0.48	5.09 ± 0.71 *^,†,‡^	<0.001
W_dec_	%	3.34 ± 1.48	4.31 ± 1.89	5.15 ± 2.36	6.34 ± 3.41 *^,†^	0.002
P_dec_	%	7.53 ± 4.15	9.14 ± 4.18	11.2 ± 4.28	12.8 ± 3.41 *^,†^	0.002

U16—age 15–15.9, U17—age 16–16.9, U18—age 17–17.9, U19—age 18–18.9. P_max_—highest value of power in 5 trials, P_mean_—mean value of highest value of power in each trial, W_tot_—total work in 5 test trials, W_max_—highest value of work in 5 trials, P_dec_—power decrement, W_dec_—work decrement. *—significantly different from U16; ^†^—significantly different from U17; ^‡^—significantly different from U18.

**Table 2:** Repeated sprint ability (RSA) test normative values with regard to playing position.

	Unit	Playing Position				
		B (n = 62)	G (n = 22)	P (n = 15)	W (n = 43)	*p*
Age	years	17.2 ± 0.93	17.3 ± 0.94	16.8 ± 0.83	17.1 ± 0.86	0.282
Height	cm	191 ± 5.47	191 ± 3.51	194 ± 6.51	184 ± 3.97	<0.001
Body mass	kg	83.5 ± 5.50	87.5 ± 6.42	99.6 ± 9.51	75.7 ± 6.29	<0.001
P_max_	W/kg	11.5 ± 0.73	11.1 ± 0.62	10.6 ± 1.02	11.8 ± 0.73	0.001
	W	957 ± 87.1	971 ± 108	1051 ± 143	896 ± 89.1	0.001
P_mean_	W/kg	10.8 ± 0.56	10.6 ± 0.48	10.1 ± 0.93	11.2 ± 0.52	<0.001
	W	904 ± 74.8	929 ± 94.3	1007 ± 131	847 ± 79.7	<0.001
W_tot_	J/kg	263 ± 19.4	259 ± 15.7	243 ± 32.4	269 ± 20.7	0.002
	kJ	22.0 ± 2.22	22.6 ± 2.29	24.2 ± 4.11	20.4 ± 2.35	<0.001
W_max_	J/kg	55.5 ± 4.73	53.8 ± 3.87	51.5 ± 7.17	56.7 ± 5.18	0.041
	kJ	4.64 ± 0.53	4.71 ± 0.54	5.14 ± 0.95	4.30 ± 0.54	0.006
W_dec_	%	5.16 ± 2.75	3.89 ± 2.02	5.56 ± 2.77	5.03 ± 2.4	0.101
P_dec_	%	11.2 ± 4.58	8.71 ± 4.02	8.00 ± 4.45	11.2 ± 4.53	0.009

B—backs, G—goalkeepers, P—pivots, W—wings. P_max_—highest value of power in 5 trials, P_mean_—mean value of highest value of power in each trial, W_tot_—total work in 5 test trials, W_max_—highest value of work in 5 trials, P_dec_—power decrement, W_dec_—work decrement.
